# Rapalink-1 Attenuates Oxidative-Stress-Induced Senescence in Vascular Cells in Association with Reduced NF-κB and MAPK Signaling

**DOI:** 10.3390/biology15090732

**Published:** 2026-05-06

**Authors:** Jinliang You, Hongjun Liu, Dilaware Khan, Majeed Rana, Sihmehmet Sahan, Katharina Faust, Sajjad Muhammad

**Affiliations:** 1 Department of Neurosurgery, Medical Faculty, University Hospital Düsseldorf, Heinrich-Heine-Universität Düsseldorf, Moorenstr. 5, 40225 Düsseldorf, Germany; jinliangyou8@gmail.com (J.Y.); hongjunliu303@gmail.com (H.L.); dilaware00@yahoo.com (D.K.); sihmehmet.sahan@med.uni-duesseldorf.de (S.S.); katharinaangela.faust@med.uni-duesseldorf.de (K.F.); 2 Department of Oral and Maxillofacial Surgery, Medical Faculty, Ulm University Hospital, Albert-Einstein-Allee 11, 89081 Ulm, Germany; majeed.rana@uni-ulm.de

**Keywords:** Rapalink-1, mTOR, oxidative stress, vascular senescence, NF-κB, MAPK, SASP

## Abstract

Oxidative stress contributes to vascular dysfunction and senescence-associated changes through activation of inflammatory and stress-responsive signaling pathways. Although the mammalian target of rapamycin (mTOR) integrates metabolic and redox-related signals, its role in vascular stress responses remains incompletely understood. In this study, we investigated the effects of Rapalink-1, an mTOR inhibitor, on H_2_O_2_-induced injury responses in human vascular endothelial cells (HUVECs) and vascular smooth muscle cells (SMCs). Oxidative stress-associated changes were assessed using oxidation-sensitive fluorescence, DNA damage markers (γ-H2AX and 8-OHDG), and senescence-associated readouts (SA-β-gal, Lamin B1, and p21). Senescence-associated secretory phenotype (SASP)-related factors were analyzed by qPCR and Western blot, and mTOR-, NF-κB-, and MAPK-related signaling was evaluated by Western blotting. H_2_O_2_ exposure reduced cell viability and increased oxidative stress-associated readouts, DNA damage markers, senescence-associated changes, and SASP-related factor expression in both HUVECs and SMCs. Rapalink-1 attenuated many of these responses, including oxidation-sensitive fluorescence, γ-H2AX and 8-OHDG staining, SA-β-gal positivity, Lamin B1 loss, p21 upregulation, and the expression of inflammatory and matrix-remodeling factors. These effects were accompanied by reduced phosphorylation of p65, p38, ERK1/2, S6, and 4EBP1. Overall, Rapalink-1 is associated with attenuation of oxidative stress-induced injury responses in vascular endothelial and smooth muscle cells, together with reduced NF-κB-, MAPK-, and mTOR-related signaling. These findings support further investigation of mTOR-targeted approaches in vascular aging and oxidative stress-related vascular dysfunction.

## Simple Summary

Oxidative stress occurs when cells are exposed to excessive reactive molecules that can damage DNA and other cellular components. In blood vessel cells, this may contribute to inflammation, dysfunction, and age-related changes. In this study, we examined whether Rapalink-1 could reduce oxidative stress-induced injury in human vascular endothelial cells and vascular smooth muscle cells. After exposure to hydrogen peroxide, both cell types showed reduced viability and increased oxidative stress-associated changes, DNA damage markers, senescence-associated features, and inflammatory and matrix-remodeling factors. Rapalink-1 attenuated many of these changes and was also associated with lower activation of stress-related signaling pathways. These findings suggest that Rapalink-1 may help reduce oxidative stress-associated injury in vascular cells and support further investigation of this pathway in vascular aging and dysfunction.

## 1. Introduction

Oxidative stress is recognized as a fundamental driver of vascular pathology and is closely implicated in cardiovascular and cerebrovascular disorders such as atherosclerosis, hypertension, stroke, and intracranial aneurysms (IAs) [1,2,3]. Among reactive oxygen species (ROS), hydrogen peroxide (H_2_O_2_) is widely used to induce experimental oxidative stress in vascular cells due to its membrane permeability [4,5]. In endothelial cells (ECs), excessive ROS decreases nitric oxide bioavailability, promotes leukocyte adhesion, and impairs vasodilatory capacity, whereas in smooth muscle cells (SMCs) it triggers phenotypic switching, matrix protease expression, and apoptosis [6,7,8,9]. Collectively, these cellular responses weaken the vascular wall, consistent with histological observations in human IA specimens, which demonstrate endothelial disruption, loss of medial SMCs, inflammatory infiltration, and extracellular matrix (ECM) disorganization [10,11,12].

In addition to reducing cell viability, oxidative stress promotes excessive intracellular ROS accumulation and disrupts redox-sensitive transcriptional programs, establishing a persistent imbalance in antioxidant defenses [4,5]. This imbalance leads to DNA lesions, including oxidized bases such as 8-OHDG and double-strand breaks marked by γ-H2AX, which activate DNA damage response signaling and accelerate premature cellular senescence [13,14,15]. Senescent vascular cells exhibit Lamin B1 loss, altered chromatin organization, and enter a state of permanent cell cycle arrest. Importantly, they also develop a senescence-associated secretory phenotype (SASP), releasing cytokines, proteases, and adhesion molecules that act in a paracrine manner to amplify inflammation and extracellular matrix degradation, thereby exacerbating vascular wall instability [16,17,18,19]. These changes impair bioenergetic function, enhance sterile inflammation, and propagate vascular injury. At the tissue level, oxidative stress also alters ECM turnover. An imbalance between matrix metalloproteinases (MMPs) and their tissue inhibitors (TIMPs) accelerates degradation of elastin and structural proteins such as Fibulin-3, leading to reduced vascular wall resilience [20,21].

At the signaling level, oxidative stress activates conserved inflammatory cascades. NF-κB integrates redox and cytokine signals to regulate chemokines, adhesion molecules, and proteases [22]. Mitogen-activated protein kinases (MAPKs), including p38 and ERK1/2, mediate stress and mitogenic responses in ECs and SMCs, linking oxidative injury to vascular remodeling [23,24,25]. The mechanistic target of rapamycin (mTOR) serves as an additional signaling hub that coordinates cellular growth, metabolism, protein synthesis, and stress adaptation [26,27,28,29]. Sustained mTOR activation has been linked to maladaptive inflammatory and stress-responsive phenotypes, whereas pharmacological inhibition of mTOR-related signaling may attenuate cellular injury responses in specific experimental contexts [27,28]. Rapalink-1, an mTOR inhibitor that combines a rapamycin-binding moiety with an ATP-competitive pharmacophore, achieves durable suppression of both complexes and has been shown to overcome resistance mechanisms encountered with earlier mTOR inhibitors in preclinical studies [30,31].

However, whether Rapalink-1 can protect vascular endothelial and smooth muscle cells—key components of the vascular wall—from oxidative stress–induced senescence remains unclear. Therefore, we employed an H_2_O_2_-induced oxidative injury model in HUVECs (a widely accepted endothelial model) and vascular smooth muscle cells (SMCs) to evaluate the protective effects of Rapalink-1. We further examined the involvement of NF-κB, MAPK, and mTOR signaling pathways, aiming to provide mechanistic insight into potential strategies for maintaining vascular stability.

## 2. Materials and Methods

### 2.1. Cell Culture

**Figure A1:**
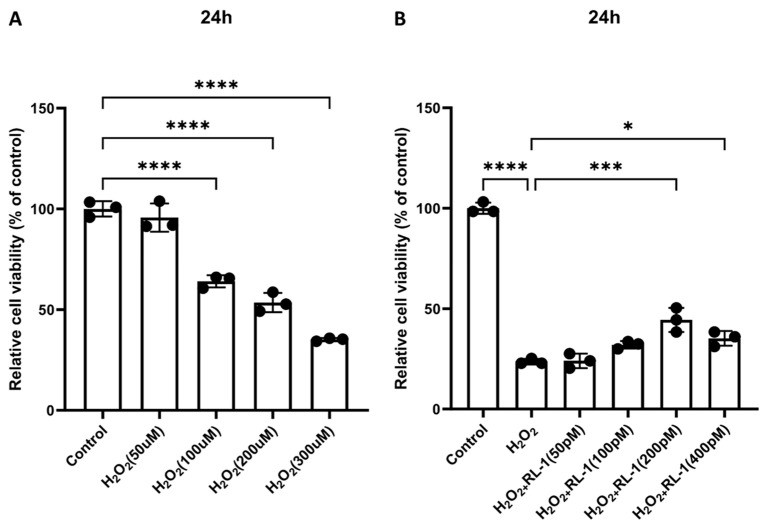
(**A**) HUVECs were treated with increasing concentrations of H_2_O_2_ (50–300 µM) for 24 h, and cell viability was assessed by MTT assay. (**B**) HUVECs were exposed to 300 µM H_2_O_2_ in the presence of increasing concentrations of Rapalink-1 (50–400 pM) for 24 h, followed by viability measurement. Data are presented as mean ± SEM (*n* = 3 independent experiments). Statistical significance was determined by one-way ANOVA with appropriate post hoc tests. Significance is indicated as: * *p*  <  0.05, *** *p*  <  0.001, **** *p*  <  0.0001.

Human umbilical-vein endothelial-cell (HUVEC) models were purchased from PromoCell (Heidelberg, Germany). HUVECs were maintained at 37 °C in a humidified incubator with 5% CO_2_ using endothelial cell medium (C-22210, PromoCell, Heidelberg, Germany) supplemented with endothelial growth factors (C-39215, PromoCell, Heidelberg, Germany). Vascular smooth muscle cell (SMC) models were likewise obtained from PromoCell (Heidelberg, Germany) and cultured at 37 °C in 5% CO_2_ in smooth muscle cell medium (C-22262) with smooth muscle growth factors (C-39267). On receipt, frozen cells were thawed and seeded into T75 culture flasks. Subculture was performed at ~80–90% confluence. For passaging, cells were rinsed with PBS and exposed to trypsin for 4 min at 37 °C under 5% CO_2_, then re-plated at 5000 cells/cm^2^ into fresh culture plates. All experiments with HUVECs and SMCs were carried out at passage 7. For treatments, cells received 300 µM hydrogen peroxide (H_2_O_2_), 200 pM Rapalink-1, or the combination of H_2_O_2_ and Rapalink-1; untreated cells served as the control. Treatment concentrations were selected based on preliminary cell-viability dose–response analyses (Appendix A, Figure A1). The 200 pM Rapalink-1 dose was chosen because it produced a reproducible effect under H_2_O_2_ exposure while remaining within a low-dose range previously used in related endothelial stress models. Each experiment was independently repeated three times using separately cultured cells, and these independent experiments were treated as biological replicates.

### 2.2. Hydrogen Peroxide (H_2_O_2_) Preparation

Hydrogen peroxide (H_2_O_2_) solution was freshly prepared in sterile water. The desired concentration was achieved by diluting the stock solution with cell culture medium. Preliminary titration experiments were performed to identify a concentration that reliably induced oxidative stress and DNA damage while preserving sufficient cell viability for downstream analyses.

### 2.3. MTT Assay

Cell viability was evaluated using the MTT (3-(4,5-dimethylthiazol-2-yl)-2,5-diphenyltetrazolium bromide) assay. HUVECs and SMCs were seeded in 96-well plates at a density of 5000 cells/cm^2^ and incubated overnight at 37 °C in a humidified atmosphere with 5% CO_2_ to allow for cell attachment. The next day, the culture medium was replaced with fresh medium containing H_2_O_2_ (300 µM), 200 pM Rapalink-1, or a combination of H_2_O_2_ and Rapalink-1. Cells cultured in drug-free medium served as the control group. After 24 h of treatment, 10 µL of MTT solution (5 mg/mL in PBS) was added to each well, and cells were incubated for an additional 3–4 h at 37 °C. Following incubation, the culture medium was carefully removed, and 100–150 µL of DMSO was added to dissolve the formazan crystals. Cell viability was assessed by measuring the optical density (OD) at 550 nm with reference at 655 nm. Background values were subtracted. All experiments were performed in triplicate.

### 2.4. DCFH-DA Staining

To assess oxidative-stress-associated fluorescence, HUVECs and SMCs were seeded at a density of 5000 cells/cm^2^ in 96-well plates. After 24 h of attachment, the culture medium was replaced with fresh medium containing either 300 µM hydrogen peroxide (H_2_O_2_), 200 pM Rapalink-1, or a combination of both agents. Medium alone served as the control. Following 2 h of exposure, cells were incubated with 10 µM 2′,7′-dichlorodihydrofluorescein diacetate (DCFH-DA; D6883, Sigma-Aldrich, St. Louis, MO, USA) for 30 min at 37 °C in the dark. After incubation, cells were rinsed three times with serum-free medium to remove residual dye. Fluorescent signals were visualized using a fluorescence microscope and quantified with ImageJ software (version 1.53c).

### 2.5. Immunofluorescence Staining

Cells were exposed to the indicated treatments (as described above) for 2 h for 8-OHDG and γ-H2AX staining, and for 24 h for Lamin B1. After treatment, cells were washed three times with PBS and fixed with 4% paraformaldehyde for 10 min at room temperature (RT). Following fixation, cells were again rinsed with PBS and permeabilized with 0.2% Triton™ X-100 for 10 min at RT. To block nonspecific binding, cells were incubated with 5% bovine serum albumin (BSA) for 1 h at RT. Subsequently, the cells were incubated overnight at 4 °C with primary antibodies against 8-OHDG, γ-H2AX, and Lamin B1 (listed in Supplementary Table S1). The next day, after three PBS washes, cells were incubated with the corresponding secondary antibodies (Supplementary Table S1) for 1 h at RT. Nuclei were counterstained with Hoechst (Sigma-Aldrich). Images were captured using a Leica (Leica Microsystems GmbH, Wetzlar, Germany) DMi8 inverted fluorescence microscope equipped with the LAS-X Life Science Microscope Software, version 3.7.5.24914 (Leica Application Suite X) and analyzed with ImageJ software (version 1.53c, National Institutes of Health, Bethesda, MD, USA). Original microscopy images are provided in the Supplementary Raw Data.

### 2.6. Western Blot

For protein analysis, HUVECs and SMCs were exposed to 300 µM hydrogen peroxide (H_2_O_2_), 200 pM Rapalink-1, or a combination of both agents for 30 min. Untreated cells served as controls. Total protein was extracted using laboratory-prepared RIPA lysis buffer, and protein concentrations were determined with the DC Protein Assay Kit (500-0116, Bio-Rad, Hercules, CA, USA). Equal amounts of protein (30 µg per sample) were resolved under reducing conditions on 12% SDS–polyacrylamide gels. Electrophoresis was carried out at 60 V for the initial 10 min and then at 90 V for an additional 60–90 min. Proteins were transferred onto 0.45 µm pore-size nitrocellulose membranes at 250 mA for 120 min. The membranes were blocked with 5% bovine serum albumin (BSA) in 0.05% TBST for 1 h at room temperature (RT) to reduce nonspecific binding, followed by overnight incubation with primary antibodies (listed in Supplementary Table S1) prepared in 5% BSA at 4 °C on a gentle shaker. After three 10 min washes with TBST, membranes were incubated with the corresponding secondary antibodies (Supplementary Table S1) diluted in 0.05% TBST for 1 h at RT. Protein bands were visualized, and densitometric analysis was performed using ImageJ software (National Institutes of Health, Bethesda, MD, USA), with protein expression normalized to GAPDH. Uncropped Western blot images are provided in the Supplementary Raw Data.

### 2.7. Quantitative Polymerase Chain Reaction (qPCR)

For qPCR analysis, HUVECs and SMCs were treated with 300 µM hydrogen peroxide (H_2_O_2_), 200 pM Rapalink-1, or a combination of both agents for 24 h. Total RNA was isolated using the NucleoSpin RNA Kit (740955.50, MACHEREY-NAGEL, Düren, Germany) according to the manufacturer’s protocol. Reverse transcription was performed with 1.2 µg of total RNA using the MMLV Reverse Transcriptase Kit (M1701, Promega, Walldorf, Germany) in the presence of Random Hexamer Primers (48190011, Thermo Fisher, Waltham, MA, USA) and RiboLock RNase Inhibitor (EO0384, Thermo Fisher). Quantitative PCR was carried out on a QuantStudio™ 3 Real-Time PCR System (Thermo Fisher Scientific, Waltham, MA, USA) using AceQ SYBR qPCR Master Mix (Q111-03, Vazyme, Nanjing, China) and gene-specific primers listed in Supplementary Table S2. The thermal cycling conditions were as follows: initial denaturation at 95 °C for 8 min, followed by 40 cycles of 95 °C for 15 s, 58.9 °C for 30 s, and 72 °C for 30 s, concluding with melting curve analysis. Relative gene expression levels were normalized to β-actin, and quantification was performed using the comparative Ct (2^−ΔΔCT^) method.

### 2.8. Senescence-Associated β-Galactosidase Staining

Cells were treated for 24 h under the conditions described above. Senescence-associated β-galactosidase activity was detected using the Senescence Cells Histochemical Staining Kit (GALS, Sigma-Aldrich, St. Louis, MO, USA) according to the manufacturer’s protocol. After treatment, cells were incubated with the SA-β-gal staining solution at 37 °C for 7 h. Following incubation, the staining solution was removed, and cells were overlaid with 70% glycerol prepared in PBS. The stained cells were then stored at 4 °C until imaging. Images were acquired using a Leica DMi8 inverted microscope equipped with the LAS-X Life Science Microscope Software (Leica Application Suite X) and analyzed with ImageJ software (version 1.53c, National Institutes of Health, Bethesda, MD, USA).

### 2.9. Statistical Analysis

Data were analyzed using GraphPad Prism (version 10.1.2) with one-way ANOVA followed by Tukey’s post hoc test.

## 3. Results

### 3.1. Rapalink-1 Enhances Vascular Cell Viability and Attenuates Oxidative Stress

To align the experimental endpoints with the expected temporal progression of H_2_O_2_-induced cellular responses, early signaling events were assessed at 30 min, oxidation-sensitive fluorescence and DNA damage-associated markers were evaluated at 2 h, and viability, transcriptional responses, senescence-associated changes, and SASP-related factors were analyzed at 24 h. This design allowed early stress signaling and oxidative injury-associated readouts to be distinguished from later phenotypic and transcriptional responses.

**Figure 1:**
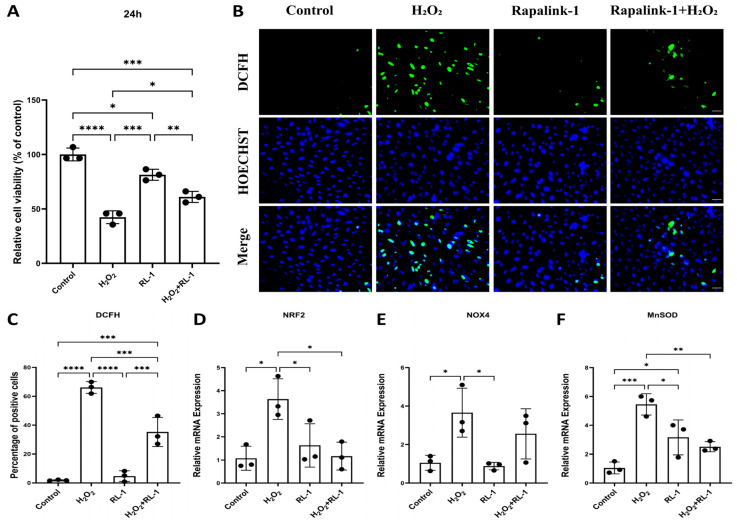
(**A**) MTT assay results in HUVECs. (**B**,**C**) Representative images of DCFH-DA staining (green), Hoechst counterstaining (blue), merged images and quantification of DCFH-DA fluorescence intensity in HUVECs. (**D**–**F**) qPCR analysis in HUVECs: *NRF2*, *NOX4*, *MnSOD*. Scale bar = 50 μm. Data are presented as mean ± SD (*n* = 3). Statistical significance was determined using one-way ANOVA with Tukey’s post hoc test. Significance is indicated as: * *p*  <  0.05, ** *p*  <  0.01, *** *p*  <  0.001, **** *p*  <  0.0001.

To determine whether Rapalink-1 modulates H_2_O_2_-induced cellular injury, we first assessed cell viability [32]. The MTT assay showed that H_2_O_2_ exposure for 24 h significantly reduced the viability of HUVECs (Figure 1A). Co-treatment with Rapalink-1 significantly attenuated this decrease.

We then examined oxidative stress-associated responses [33]. DCFH-DA staining showed that H_2_O_2_ markedly increased intracellular oxidation-sensitive fluorescence in HUVECs (Figure 1B,C), whereas co-treatment with Rapalink-1 significantly reduced this signal, indicating attenuation of oxidation-sensitive cellular responses. Consistent with these findings, qPCR analysis demonstrated that the H_2_O_2_-induced upregulation of redox-associated genes, including *NRF2*, *NOX4*, and *MnSOD,* was attenuated by Rapalink-1 (Figure 1D–F). Similar effects on cell viability and oxidative stress-associated readouts were observed in SMCs (Supplementary Figure S1).

### 3.2. Rapalink-1 Attenuates H_2_O_2_-Induced DNA Damage-Associated Changes in Vascular Cells

**Figure 2:**
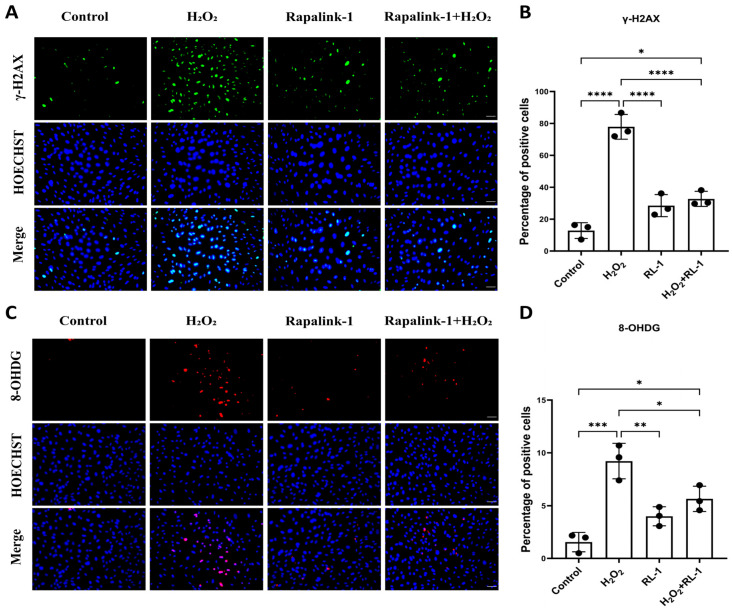
(**A**,**B**) Representative immunofluorescence images and quantification of γ-H2AX (green) in HUVECs with Hoechst nuclear counterstain (blue) and merged images. (**C**,**D**) Representative images and quantification of 8-OHDG (red) in HUVECs with Hoechst nuclear counterstain (blue) and merged images. Scale bar = 50 μm. Data are presented as mean ± SD (*n* = 3). Statistical significance was determined using one-way ANOVA with Tukey’s post hoc test. Significance is indicated as: * *p*  <  0.05, ** *p*  <  0.01, *** *p*  <  0.001, **** *p*  <  0.0001.

We then examined whether H_2_O_2_-induced oxidative injury was accompanied by DNA damage in vascular cells. In HUVECs, H_2_O_2_ markedly increased the percentage of γ-H2AX-positive nuclei relative to control (47.88 ± 6.58% vs. 9.81 ± 0.50%, *p* < 0.0001), and this increase was significantly attenuated by co-treatment with Rapalink-1 (17.86 ± 3.17%, *p* < 0.001 vs. H_2_O_2_) (Figure 2A,B). 8-OHDG staining showed a similar pattern. H_2_O_2_ increased 8-OHDG positivity from 16.55 ± 2.48% in control cells to 37.40 ± 5.05% (*p* < 0.0001), whereas Rapalink-1 significantly reduced this response (29.21 ± 1.83%, *p* < 0.05 vs. H_2_O_2_) (Figure 2C,D). Similar findings were obtained in SMCs, where Rapalink-1 also reduced H_2_O_2_-induced γ-H2AX and 8-OHDG staining (Supplementary Figure S2).

### 3.3. Rapalink-1 Attenuates Oxidative Stress-Induced Senescence-Associated Changes in Vascular Cells

Senescence-associated changes in HUVECs and SMCs were evaluated using established markers under oxidative stress conditions [34,35].

**Figure 3:**
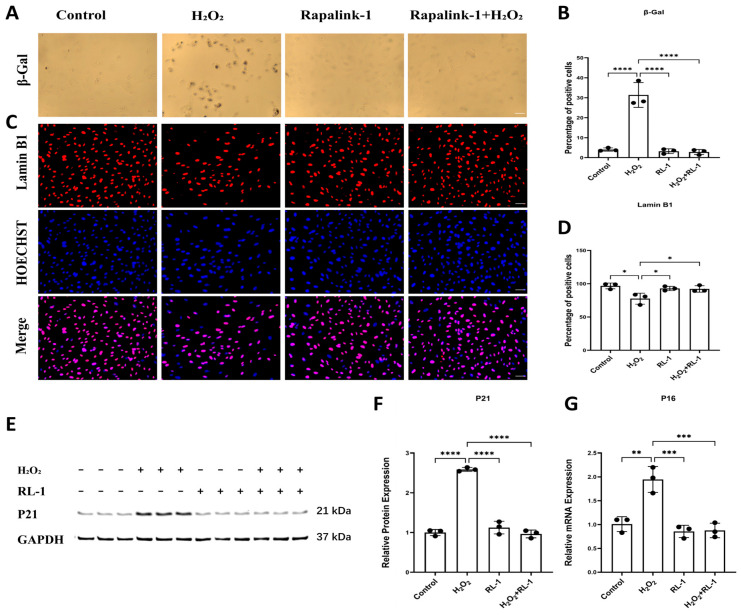
(**A**,**B**) Representative images and quantification of SA-β-gal staining in HUVECs. (**C**,**D**) Representative immunofluorescence images and quantification of Lamin B1 (red) in HUVECs with Hoechst counterstain (blue) and merged images. (**E**,**F**) Representative Western blots and quantification of the senescence-associated cell cycle regulator p21 in HUVECs. (**G**) qPCR analysis of *p16* mRNA expression in HUVECs. Protein expression was normalized to GAPDH. Scale bar = 50 μm. Data are presented as mean ± SD (*n* = 3). Statistical significance was determined using one-way ANOVA with Tukey’s post hoc test. Significance is indicated as: * *p*  <  0.05, ** *p*  <  0.01, *** *p*  <  0.001, **** *p*  <  0.0001.

In HUVECs, H_2_O_2_ exposure was accompanied by a marked increase in SA-β-gal-positive cells relative to control (31.39 ± 6.23% vs. 4.06 ± 0.82%, *p* < 0.0001), and this increase was significantly attenuated by Rapalink-1 co-treatment (2.82 ± 1.28%, *p* < 0.0001 vs. H_2_O_2_) (Figure 3A,B). A similar pattern was observed for Lamin B1 staining, with H_2_O_2_ significantly decreasing the proportion of Lamin B1-positive nuclei (77.51 ± 8.23% vs. 96.65 ± 4.31% in control, *p* = 0.0121), whereas Rapalink-1 significantly alleviated this effect (92.17 ± 4.85%, *p* < 0.05 vs. H_2_O_2_) (Figure 3C,D).

Consistently, H_2_O_2_ markedly increased p21 protein levels (2.59 ± 0.05 vs. 1.00 ± 0.08 in control, *p* < 0.0001), while Rapalink-1 significantly reduced p21 accumulation (0.96 ± 0.10, *p* < 0.0001 vs. H_2_O_2_); Rapalink-1 alone did not significantly affect basal p21 expression (Figure 3E,F).

To further clarify the p16 expression status, p16 mRNA expression was assessed by qPCR. H_2_O_2_ exposure increased p16 expression, whereas Rapalink-1 co-treatment attenuated this response (Figure 3G). Together with SA-β-gal staining, Lamin B1 loss, and p21 upregulation, these findings support the attenuation of H_2_O_2_-induced senescence-associated changes by Rapalink-1. Similar changes were observed in SMCs, where Rapalink-1 was likewise associated with preservation of Lamin B1 and lower p21 and p16 expression under oxidative stress conditions (Supplementary Figure S3).

### 3.4. Rapalink-1 Attenuates SASP-Related Inflammatory, Cytokine, Adhesion-Related, and Matrix-Remodeling Factors in Vascular Cells

**Figure 4:**
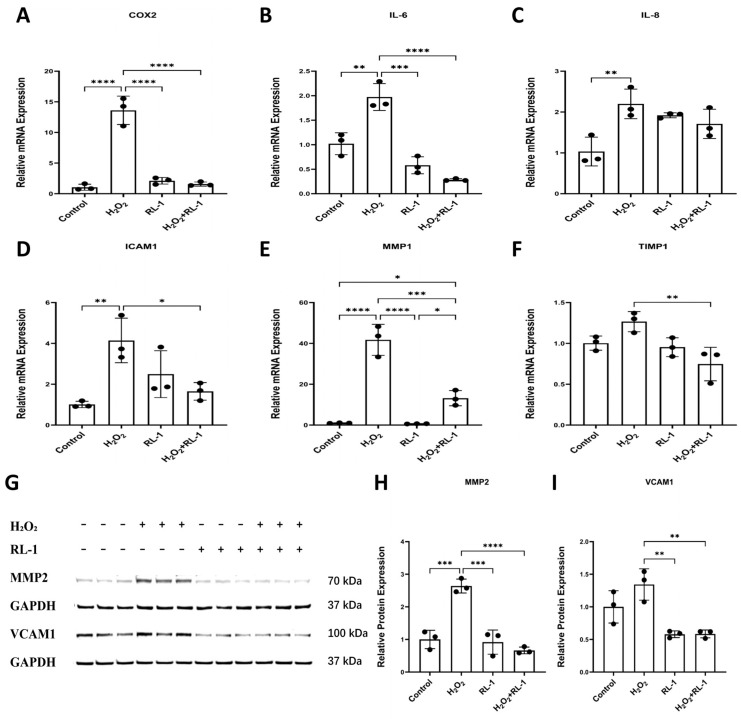
(**A**–**F**) qPCR analysis of SASP-related inflammatory, cytokine, adhesion-related, and matrix-remodeling factors in HUVECs, including *COX2*, *IL-6*, *IL-8*, *ICAM1*, *MMP1*, and *TIMP1*. (**G**–**I**) Representative Western blots and quantification of relative protein expression of MMP2 and VCAM1 in HUVECs. Protein expression was normalized to GAPDH. Data are presented as mean ± SD (*n* = 3). Statistical significance was determined using one-way ANOVA with Tukey’s post hoc test. Significance is indicated as: * *p*  <  0.05, ** *p*  <  0.01, *** *p*  <  0.001, **** *p*  <  0.0001.

H_2_O_2_ exposure was accompanied by increased expression of several SASP-related inflammatory, cytokine, adhesion-related, and matrix-remodeling factors in HUVECs, including *COX2*, *IL-6*, *IL-8*, *ICAM1*, *MMP1*, and *TIMP1* (Figure 4A–F). Rapalink-1 alone did not substantially alter the basal expression of most of these genes, although *IL-8* showed a less uniform pattern under the present conditions. Under H_2_O_2_ exposure, Rapalink-1 attenuated the induction of several SASP-related factors, with a clearer effect observed on *IL-6* than on *IL-8* in HUVECs. Consistent with the transcriptional data, H_2_O_2_ also increased MMP2 and VCAM1 protein abundance in HUVECs, and both were reduced by Rapalink-1 co-treatment (Figure 4G–I). In SMCs, Rapalink-1 was associated with lower expression of several H_2_O_2_-induced SASP-related factors, including *IL-6*, *IL-8*, *COX2*, *MMP1*, and *TIMP1*, although the response pattern was not identical across all markers (Supplementary Figure S4).

### 3.5. Rapalink-1 Is Associated with Reduced NF-κB, MAPK, and mTOR-Related Signaling

Consistently, exposure to H_2_O_2_ triggered robust phosphorylation of NF-κB p65, p38 MAPK, and ERK1/2 in both HUVECs and SMCs. In parallel, growth-related mTOR signaling was engaged, as evidenced by increased phosphorylation of ribosomal protein S6 [28,29].

**Figure 5:**
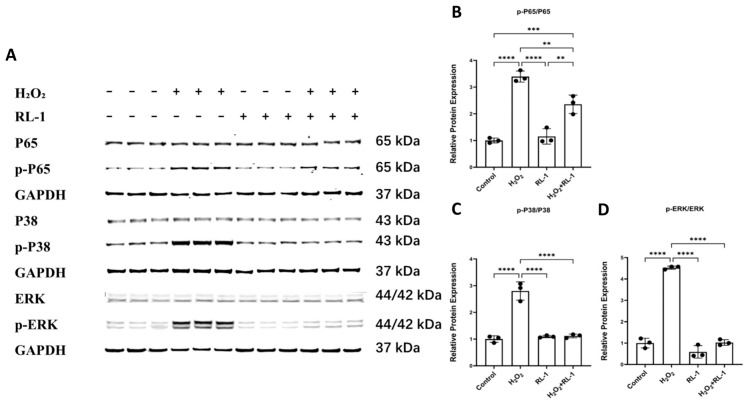
(**A**) Representative Western blot images showing the protein levels of p65, p-p65, p38, p-p38, ERK, and p-ERK (HUVECs). (**B**–**D**) Quantitative analysis of p-p65/p65, p-p38/p38, and p-ERK/ERK (HUVECs). Protein expression was normalized to GAPDH. Data are presented as mean ± SD (*n* = 3). Statistical significance was determined using one-way ANOVA with Tukey’s post hoc test. Significance is indicated as: ** *p*  <  0.01, *** *p*  <  0.001, **** *p*  <  0.0001.

In HUVECs, H_2_O_2_ exposure strongly activated the NF-κB and MAPK signaling pathways, as evidenced by significantly increased phosphorylation ratios of p65 (p-p65/p65), p38 (p-p38/p38), and ERK (p-ERK/ERK) (Figure 5A–D). Co-treatment with Rapalink-1 markedly attenuated these increases, restoring the phosphorylation levels of p65, p38, and ERK near baseline levels. Notably, Rapalink-1 did not significantly alter the total protein abundance of these signaling molecules, indicating that its inhibitory effect is specifically targeted at their activation state.

A parallel analysis in SMCs confirmed that Rapalink-1 similarly suppressed H_2_O_2_-induced activation of these pathways (Supplementary Figure S5).

**Figure 6:**
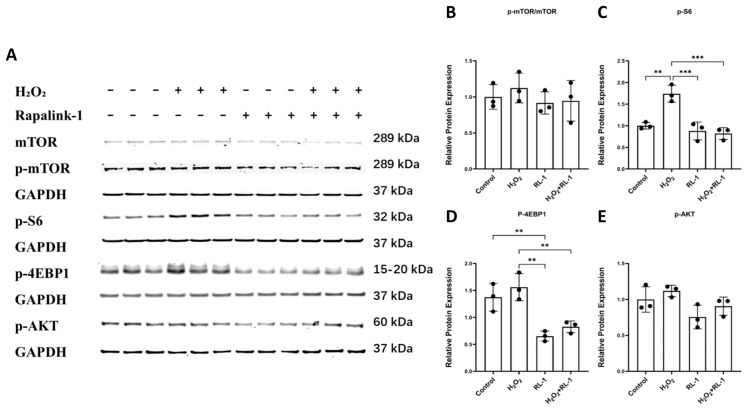
(**A**) Representative Western blot images showing the protein levels of mTOR, p-mTOR, p-S6, p-4EBP1 (HUVECs). (**B**–**E**) Quantitative analysis of p-mTOR/mTOR, p-S6, p-4EBP1, p-AKT (HUVECs). Protein expression was normalized to GAPDH. Data are presented as mean ± SD (*n* = 3). Statistical significance was determined using one-way ANOVA with Tukey’s post hoc test. Significance is indicated as: ** *p*  <  0.01, *** *p*  <  0.001.

mTOR-related signaling was then examined in HUVECs and SMCs under H_2_O_2_ exposure. In HUVECs, the p-mTOR/mTOR ratio was not significantly altered by H_2_O_2_ treatment (Figure 6A,B). In contrast, SMCs showed a significant increase in p-mTOR/mTOR under H_2_O_2_ stimulation, which was attenuated by Rapalink-1 co-treatment (Supplementary Figure S6). Analysis of downstream targets in HUVECs showed that p-S6 changed only modestly, whereas p-4EBP1 was significantly increased following H_2_O_2_ exposure (Figure 6A,C,D). Rapalink-1 reduced phosphorylation of both S6 and 4EBP1. To further assess mTORC2-related signaling, p-AKT was also examined. In HUVECs, p-AKT did not show a pronounced treatment-related difference under the present experimental conditions (Figure 6A,E).

In SMCs, H_2_O_2_ also increased phosphorylation of S6, 4EBP1, and AKT, and these responses were reduced by Rapalink-1 (Supplementary Figure S6C–E). These data support an association between Rapalink-1 treatment and attenuation of mTOR-related downstream signaling in vascular cells under oxidative stress conditions, with a more evident AKT-related response in SMCs than in HUVECs.

## 4. Discussion

The present study shows that Rapalink-1 is associated with attenuation of injury responses caused by oxidative stress in HUVECs and SMCs. Across both vascular cell types, Rapalink-1 reduced the loss of viability induced by H_2_O_2_, decreased readouts related to oxidative stress, limited the accumulation of DNA damage markers, and attenuated changes related to senescence and the senescence-associated secretory phenotype. These effects were accompanied by lower NF-κB and MAPK signaling and by suppression of downstream mTOR targets. These findings suggest that Rapalink-1 modulates mTOR-related signaling within a broader network of stress responses that influences how vascular cells respond to acute oxidative injury [36,37,38].

An important observation of this study is that Rapalink-1 was associated with a reduction in cellular responses related to oxidative stress. In our system, H_2_O_2_ increased oxidation-sensitive fluorescence and altered the expression of redox-associated genes, including NOX4, MnSOD, and NRF2, whereas these redox-associated responses were attenuated by Rapalink-1. Because acute H_2_O_2_ exposure triggers both oxidative injury and compensatory antioxidant responses, the parallel reduction in oxidation-sensitive fluorescence and redox-responsive transcription is most consistent with an overall decrease in stress burden rather than a simple suppression of defense pathways [4,5,20,39,40]. This interpretation does not distinguish between a direct antioxidant effect and secondary changes arising from altered cellular signaling. At the same time, the present data do not resolve the subcellular source of ROS, and our conclusions therefore remain limited to the level of overall cellular responses associated with oxidative stress. This distinction is important because redox signaling in vascular cells is highly compartmentalized and may have different consequences depending on cellular context [4,5].

The reduction in oxidative stress-related readouts was accompanied by attenuation of DNA damage markers and changes associated with senescence. Exposure to H_2_O_2_ increased γ-H2AX and 8-OHDG staining, increased SA-β-gal staining, and elevated p21-and p16-associated senescence readouts, whereas Rapalink-1 attenuated these changes. These findings are compatible with the idea that mTOR-related signaling contributes to senescence-associated stress responses under conditions of oxidative stress. Previous work has linked oxidative DNA damage to endothelial dysfunction, vascular aging, and the establishment of stable growth arrest programs [13,14,41,42,43,44,45,46]. In this context, our data suggest that Rapalink-1 may act upstream of, or in parallel with, these processes by limiting the cellular consequences of oxidative injury rather than by targeting a single downstream effector of senescence. Because p53 was not directly assessed, the present study does not allow firm conclusions about specific checkpoint hierarchies. However, the coordinated reduction in DNA damage- and senescence-associated markers is consistent with the view that mTOR-related signaling participates in the progression from acute oxidative stress toward a more persistent injury phenotype.

Another relevant finding is the attenuation of gene and protein expression related to the senescence-associated secretory phenotype. Oxidative stress increased transcripts linked to inflammation, cytokine signaling, adhesion, and matrix remodeling, including *COX2*, *IL-6*, *IL-8*, *ICAM1*, *MMP1*, and *TIMP1*, and also increased the abundance of VCAM1 and MMP2 proteins. Rapalink-1 reduced a substantial proportion of these responses, although the cytokine-related pattern was not entirely uniform across all markers and cell types. This is potentially important because the vascular consequences of senescence are not limited to cell cycle arrest. They also involve paracrine amplification of inflammation, leukocyte recruitment, and extracellular matrix remodeling through mediators associated with the senescence-associated secretory phenotype [16,18,19,36,47,48,49]. From this perspective, Rapalink-1 may be relevant not only because it attenuates markers of senescence within individual cells, but also because it reduces the expression of SASP-related factors associated with inflammatory and matrix-remodeling responses. The TIMP1 response was less uniform than that of the other transcripts, suggesting that Rapalink-1 does not simply normalize all stress-responsive genes in parallel. Rather, it may compress the overall amplitude of the transcriptional program associated with stress while leaving some extracellular matrix-related components differentially affected. Given the context-dependent roles of TIMPs in vascular remodeling, this point warrants cautious interpretation and would require more detailed functional analysis [20,21,49].

At the signaling level, the data are consistent with coordinated modulation of NF-κB, MAPK, and mTOR-related pathways. The NF-κB and MAPK cascades are well-established mediators of inflammatory gene expression, stress adaptation, and regulation of the senescence-associated secretory phenotype under oxidative conditions [22,23,24,25,50,51]. In the present study, H_2_O_2_ increased phosphorylation of p65, p38, and ERK1/2, and these changes were attenuated by Rapalink-1. Because these pathways lie at the interface of inflammatory transcription and stress signaling, their attenuation provides a plausible context for interpreting the broader reduction in DNA damage- and SASP-related responses observed after Rapalink-1 treatment. However, our data are correlative in this regard and do not establish the order of these pathways. It therefore remains possible that suppression of NF-κB and MAPK signaling reflects reduced upstream cellular stress rather than a primary direct effect of Rapalink-1 on these pathways.

The mTOR-related readouts showed a somewhat different pattern. In HUVECs, the p-mTOR/mTOR ratio was not significantly altered by H_2_O_2_, whereas phosphorylation of the downstream targets S6 and 4E-BP1 was reduced by Rapalink-1. In SMCs, by contrast, H_2_O_2_ increased both the p-mTOR/mTOR ratio and phosphorylation of downstream targets, and these responses were attenuated by Rapalink-1. This pattern suggests that downstream signaling readouts may be more informative than p-mTOR alone in this experimental setting, which is in line with earlier reports indicating that pathway activity is not always adequately captured by a single upstream phosphorylation marker [26,27,28,29]. The additional AKT analysis further refined this interpretation. Whereas p-AKT did not show a pronounced treatment-related difference in HUVECs under the present conditions, SMCs showed a clearer H_2_O_2_-associated increase in p-AKT that was attenuated by Rapalink-1. This apparent difference may reflect cell-specific integration of stress signals, which is biologically plausible given that endothelial cells and vascular smooth muscle cells occupy distinct metabolic and signaling states within the vessel wall and contribute differently to vascular adaptation and disease [7,17,19]. Our results therefore indicate that Rapalink-1 is associated with reduced downstream mTOR-related signaling in both cell types, while the AKT-related response was more evident in SMCs than in HUVECs.

These findings may be relevant to vascular disorders in which oxidative stress, inflammation, and maladaptive remodeling are central pathogenic features. In conditions such as intracranial aneurysm and other forms of vascular wall degeneration, endothelial dysfunction, inflammatory activation, extracellular matrix disorganization, and loss of smooth muscle cells are thought to interact in a self-reinforcing manner [10,11,12,40]. Within that framework, attenuation of injury associated with oxidative stress, inflammatory signaling, and matrix remodeling factors by Rapalink-1 is biologically relevant. Nevertheless, the present data should not be interpreted as evidence that Rapalink-1 is disease-modifying in vivo. Our experiments were performed in simplified cell culture systems under conditions of acute oxidative stress and therefore address only selected components of a much more complex disease process. The current findings are better viewed as mechanistic support for the broader idea that mTOR-related signaling participates in vascular injury responses induced by stress and may represent a tractable node for therapeutic intervention.

The potential translational relevance of these findings should be considered within the broader biological roles of mTOR. mTOR is a central regulator of cellular growth, metabolism, protein synthesis, and inflammatory stress responses, and its inhibition can have context-dependent effects across tissues [26,27,28,29,38]. Rapalink-1 was developed to achieve potent and sustained target engagement, including activity against resistance-associated states observed with earlier mTOR inhibitors [30,52,53]. This pharmacological profile may be advantageous when persistent mTOR signaling contributes to maladaptive stress responses. At the same time, deeper pathway inhibition may also increase the risk of physiological consequences outside the intended target context, especially in tissues in which mTOR activity is required for repair, survival, or barrier function. Any translational development would therefore require careful evaluation of dose, timing of intervention, vascular bed specificity, and systemic safety. The present co-treatment design addresses attenuation of injury responses during oxidative stress exposure, whereas post-treatment or rescue paradigms will be required to determine whether Rapalink-1 can reverse already established stress responses. In this regard, our data support further preclinical investigation, but they do not yet define the therapeutic window or predict net benefit in vivo.

These findings also provide a useful cellular framework for considering stress-related vascular injury in broader vascular contexts. Oxidative stress, inflammatory signaling, vascular cell senescence, and matrix remodeling are common processes shared by several vascular disorders, including cerebrovascular disease [54,55,56]. In this regard, the present data are relevant because they show that Rapalink-1 attenuates multiple injury-associated responses in two major vascular cell types, endothelial cells and smooth muscle cells. At the same time, disease-specific relevance will require further validation in cerebrovascular or pathology-specific models.

Several limitations should be acknowledged. The study was performed in vitro and used an acute H_2_O_2_ model, which does not fully recapitulate the complexity of vascular disease in vivo. In addition, although NF-κB, MAPK, and mTOR-related signaling changed in parallel with the observed phenotypic effects, the present data do not establish a direct causal hierarchy among these pathways. Further studies in chronic and in vivo models will be required to better define the vascular relevance and translational potential of Rapalink-1.

In summary, our findings support an association between Rapalink-1 treatment and attenuation of oxidative stress-induced injury responses in vascular endothelial and smooth muscle cells. Rather than indicating a single dominant mechanism, the data point to coordinated modulation of oxidative stress-associated, inflammatory, senescence-related, and mTOR-dependent processes. This integrative effect may be relevant to vascular conditions characterized by chronic stress signaling and maladaptive remodeling, and it provides a rationale for further mechanistic and translational studies of mTOR-targeted strategies in vascular aging and disease.

## 5. Conclusions

In conclusion, Rapalink-1 treatment was associated with attenuation of H_2_O_2_-induced injury responses in HUVECs and SMCs. These responses included oxidative stress-associated readouts, DNA damage marker accumulation, senescence-associated changes, and expression of factors related to the senescence-associated secretory phenotype. These changes were accompanied by reduced NF-κB-, MAPK-, and mTOR-related signaling. The findings support further investigation of mTOR-directed strategies in vascular aging and oxidative stress-associated vascular dysfunction.

## Data Availability

The data presented in this study are available on request from the corresponding author.

## Supplementary Materials

The following supporting information can be downloaded at: https://www.mdpi.com/article/10.3390/biology15090732/s1. Supplementary Figures S1–S6: Related figures in SMCs; Supplementary Raw Data: Original microscopy images; Uncropped Western blot images. Supplementary Tables: Table S1 (List of primary and secondary antibodies used in this study); Table S2 (Primer sequences used for qRT-PCR).

## Abbreviations

The following abbreviations are used in this manuscript:

8-OHDG	8-hydroxy-2′-deoxyguanosine
BSA	bovine serum albumin
cDNA	complementary DNA
COX2	cyclooxygenase-2
DCFH-DA	2′,7′-dichlorodihydrofluorescein diacetate
ECs	endothelial cells
ERK	extracellular signal-regulated kinase (ERK1/2)
GAPDH	glyceraldehyde-3-phosphate dehydrogenase
γ-H2AX	phosphorylated histone H2AX
HO-1	heme oxygenase-1
HUVECs	human umbilical vein endothelial cells
IA	intracranial aneurysm
ICAM1	intercellular adhesion molecule-1
IF	immunofluorescence
IL-6	interleukin-6
IL-8	interleukin-8
Lamin B1	nuclear lamin B1
MAPK	mitogen-activated protein kinase
MnSOD	manganese superoxide dismutase (SOD2)
MMP1	matrix metalloproteinase 1
mTOR	mechanistic target of rapamycin
MTT	3-(4,5-dimethylthiazol-2-yl)-2,5-diphenyltetrazolium bromide
NF-κB	nuclear factor kappa-light-chain-enhancer of activated B cells
NOX4	NADPH oxidase 4
NRF2	nuclear factor erythroid 2–related factor 2 (NFE2L2)
p16	cyclin-dependent kinase inhibitor 2A
p21	cyclin-dependent kinase inhibitor 1
p38	p38 mitogen-activated protein kinase (MAPK14)
p-4EBP1	phosphorylated eukaryotic translation initiation factor 4E-binding protein 1
p65	nuclear factor kappa-light-chain-enhancer of activated B cells p65
p-AKT	phosphorylated AKT (Ser473)
RIPA	radioimmunoprecipitation assay (buffer)
ROS	reactive oxygen species
RT	room temperature
SA-β-gal	senescence-associated β-galactosidase
SASP	senescence-associated secretory phenotype
SMCs	smooth muscle cells (vascular)
SOD1	superoxide dismutase 1
S6	ribosomal protein S6
TBST	tris-buffered saline with Tween-20
TIMP1	tissue inhibitor of metalloproteinases 1
VCAM1	vascular cell adhesion molecule 1
