# Supplementary material for: Rapalink-1 Attenuates Oxidative-Stress-Induced Senescence in Vascular Cells in Association with Reduced NF-κB and MAPK Signaling
Source: Biology (Basel). 2026 May 6;15(9):732. doi: 10.3390/biology15090732 (PMC13162792; doi:10.3390/biology15090732)
Supplement: Supplementary file 1 [file biology-15-00732-s001.zip › Supplementary Figure.pdf]

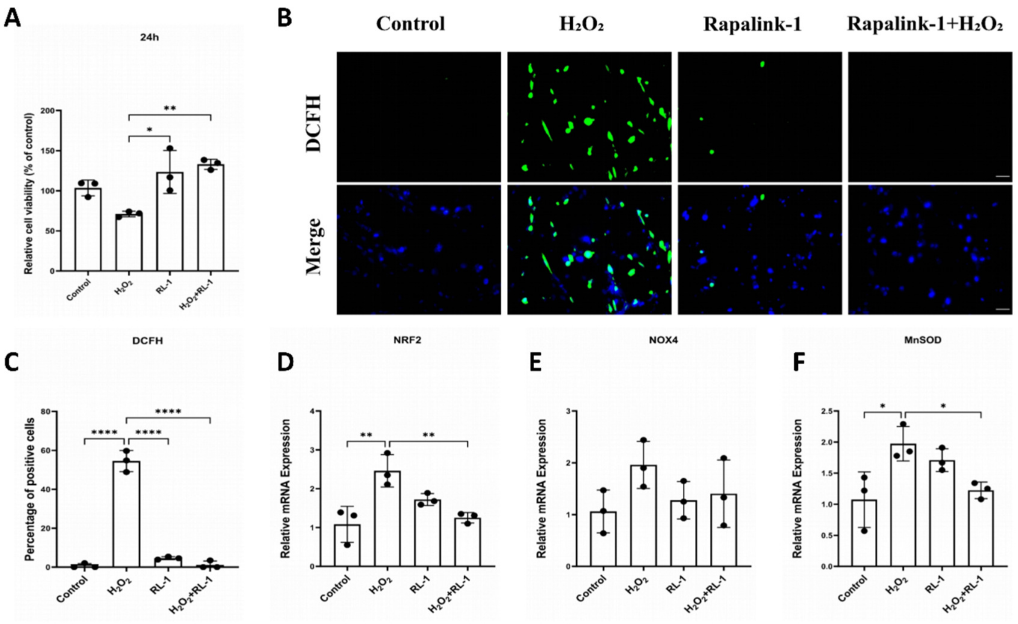

Figure S1. (A) Cell viability assessed by MTT assay at 24 h. (B) Representative images of DCFH-DA staining. (C) Quantification of intracellular ROS levels measured by DCFH-DA staining. (D–F) qPCR analysis in SMCs: NRF2, NOX4, MnSOD. Scale bar = 50 μm. Data are presented as mean ± SD (n = 3). Statistical significance was determined using one-way ANOVA with Tukey's post hoc test. Significance is indicated as: *$p < 0.05$, **$p < 0.01$, ***$p < 0.001$, ****$p < 0.0001$.

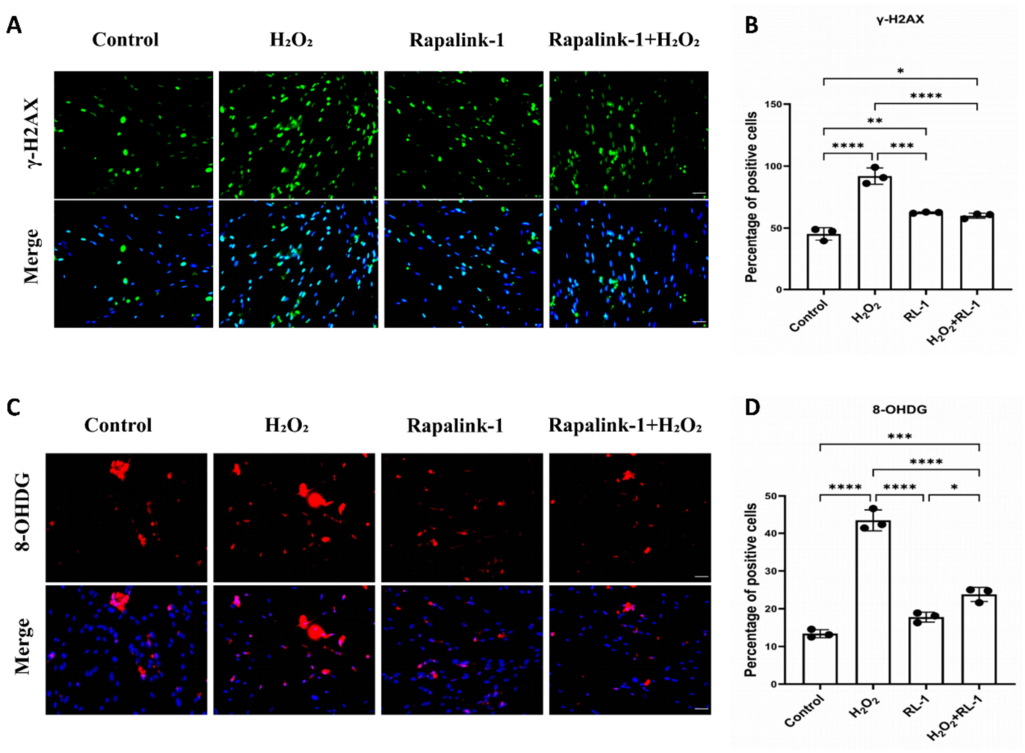

Figure S2. (A, B) Representative immunofluorescence images and quantification of γ-H2AX in SMCs. (C, D) Representative images and quantification of 8-OHDG in SMCs. Scale bar = 50 μm. Data are presented as mean ± SD (n = 3). Statistical significance was determined using one-way ANOVA with Tukey's post hoc test. Significance is indicated as: $^{*}p < 0.05$, $^{**}p < 0.01$, $^{***}p < 0.001$, $^{****}p < 0.0001$.

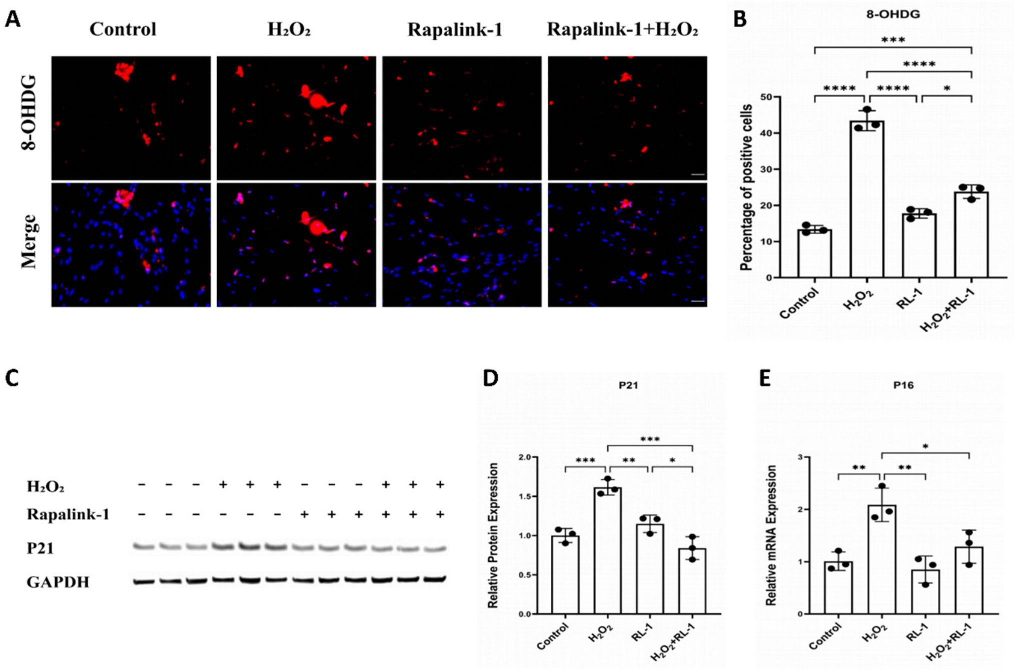

Figure S3. (A, B) Representative immunofluorescence images and quantification of Lamin B1 in SMCs. (C, D) Representative western blots and quantification of the senescence-associated cell cycle regulator P21 in SMCs. (E) qPCR analysis of p16 mRNA expression in SMCs. Protein expression was normalized to GAPDH. Scale bar = 50 μm. Data are presented as mean ± SD ($n = 3$). Statistical significance was determined using one-way ANOVA with Tukey's post hoc test. Significance is indicated as: *$p < 0.05$, **$p < 0.01$, ***$p < 0.001$, ****$p < 0.0001$. Note: SA-β-gal staining was not reliably induced in SMCs under these conditions; thus, senescence was evaluated using P21, Lamin B1.

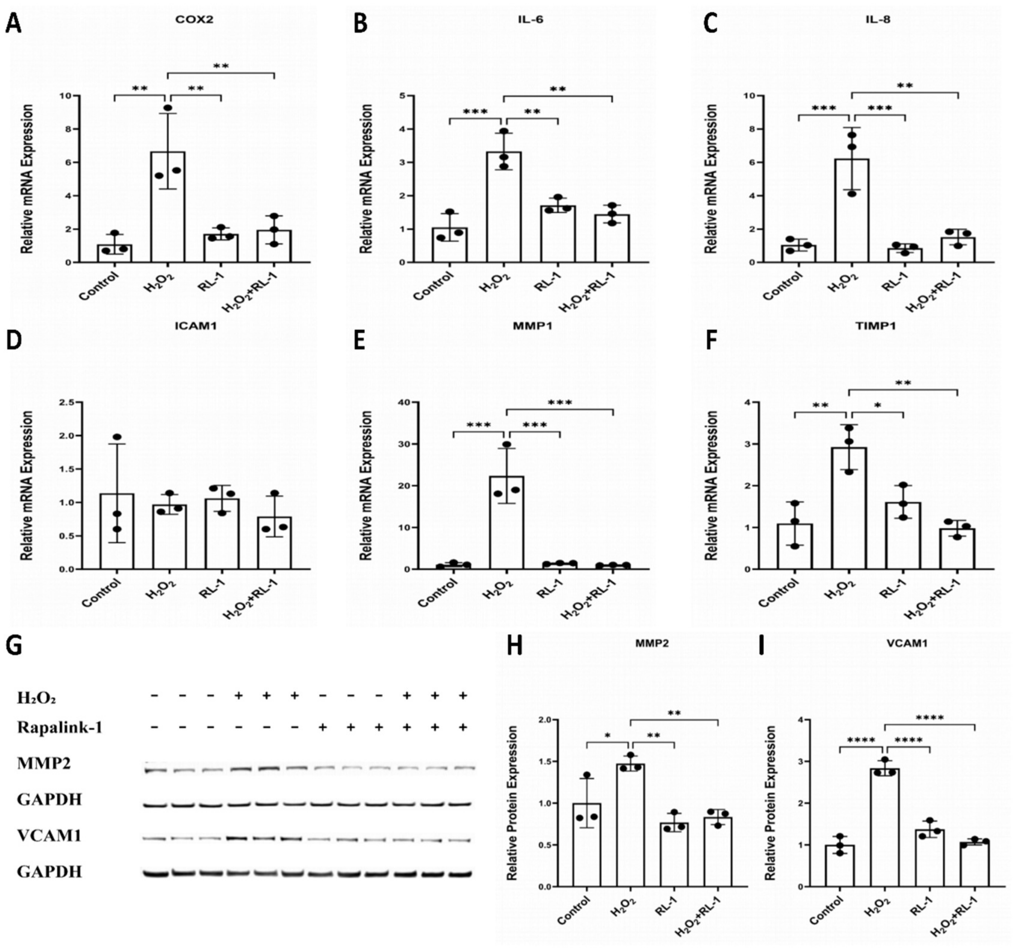

Figure S4. (A–F) qPCR analysis of SASP-related genes in SMCs, including COX2, IL-6, IL-8, ICAM1, MMP1, and TIMP1. (G–I) Representative Western blots and quantification of relative protein expression of MMP2 and VCAM1 in SMCs. Protein expression was normalized to GAPDH. Data are presented as mean ± SD ($n = 3$). Statistical significance was determined using one-way ANOVA with Tukey's post hoc test. Significance is indicated as: *$p < 0.05$, **$p < 0.01$, ***$p < 0.001$, ****$p < 0.0001$.

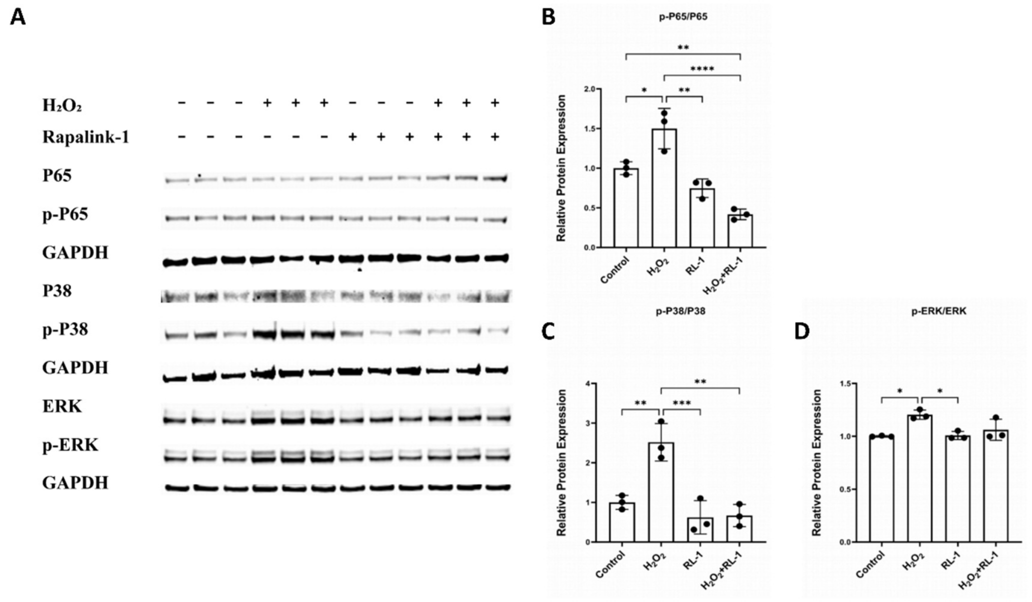

Figure S5. (A) Representative Western blot images showing the protein levels of P65, p-P65, P38, p-P38, ERK, and p-ERK in SMCs. (B–D) Quantitative analysis of p-P65/P65, p-P38/P38, and p-ERK/ERK in SMCs. Protein expression was normalized to GAPDH. Data are presented as mean ± SD (n = 3). Statistical significance was determined using one-way ANOVA with Tukey's post hoc test. Significance is indicated as: $^{*}p < 0.05$, $^{**}p < 0.01$, $^{***}p < 0.001$, $^{****}p < 0.0001$.

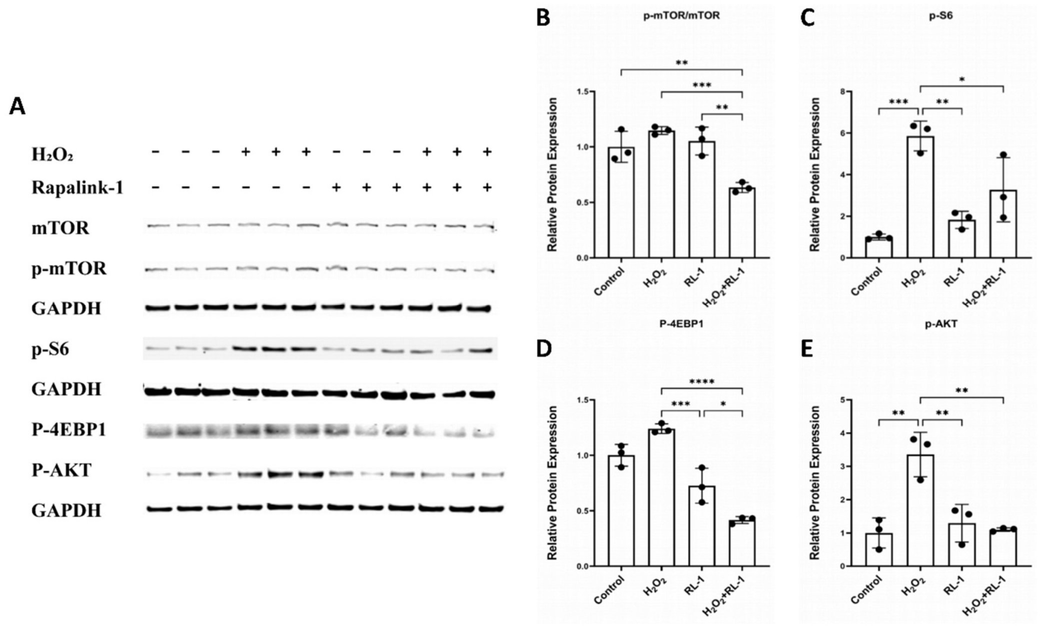

Figure S6. (A) Representative Western blot images showing the protein levels of mTOR, p-mTOR, p-S6, p-4EBP1, and p-AKT in SMCs. (B–E) Quantitative analysis of p-mTOR/mTOR, p-S6, p-4EBP1, and p-AKT in SMCs. Protein expression was normalized to GAPDH. Data are presented as mean ± SD (n = 3). Statistical significance was determined using one-way ANOVA with Tukey's post hoc test. Significance is indicated as: *$p < 0.05$, **$p < 0.01$, ***$p < 0.001$, ****$p < 0.0001$.
