# Supplementary material for: Rapalink-1 Attenuates Oxidative-Stress-Induced Senescence in Vascular Cells in Association with Reduced NF-κB and MAPK Signaling
Source: Biology (Basel). 2026 May 6;15(9):732. doi: 10.3390/biology15090732 (PMC13162792; doi:10.3390/biology15090732)

Representative immunofluorescence images corresponding to Figure 1B.

Figure 1 DCFH OF HUVECs

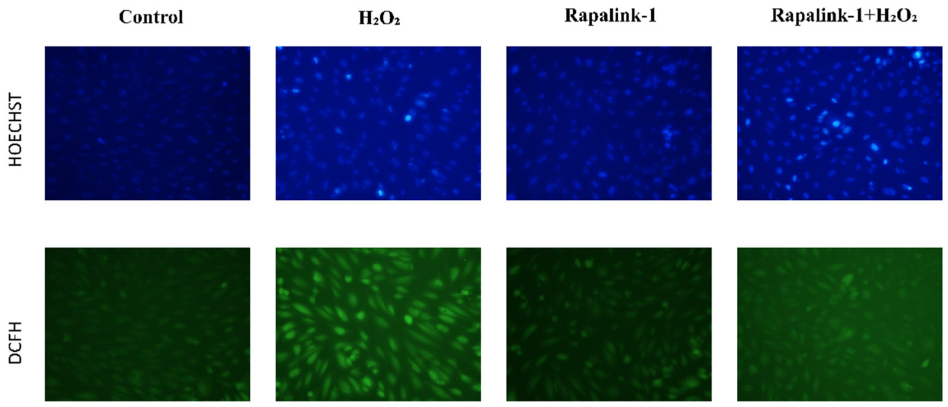

Representative immunofluorescence images corresponding to Supplementary Figure S1 A

Figure S1 DCFH OF SMCs

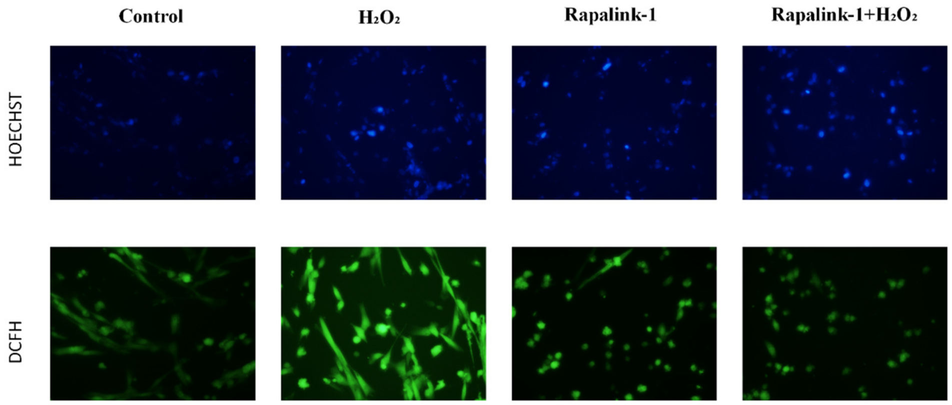

Representative immunofluorescence images corresponding to Figure 2 A, C

Figure 2 γ-H2AX OF HUVECS

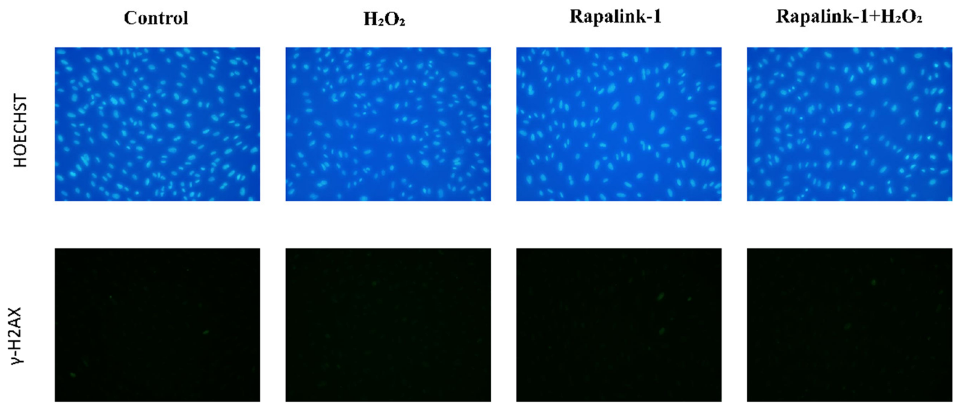

Figure 2 8-OHDG OF HUVECS

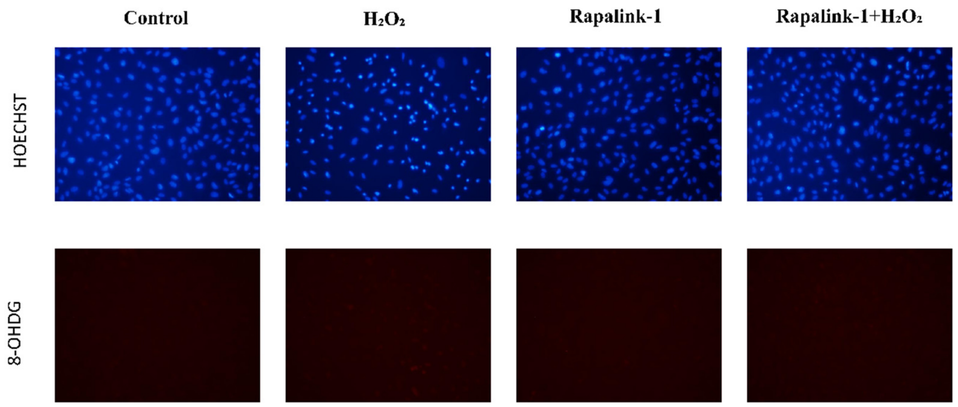

Representative immunofluorescence images corresponding to Supplementary Figure S2A, C

Figure S2 A γ-H2AX OF SMCS

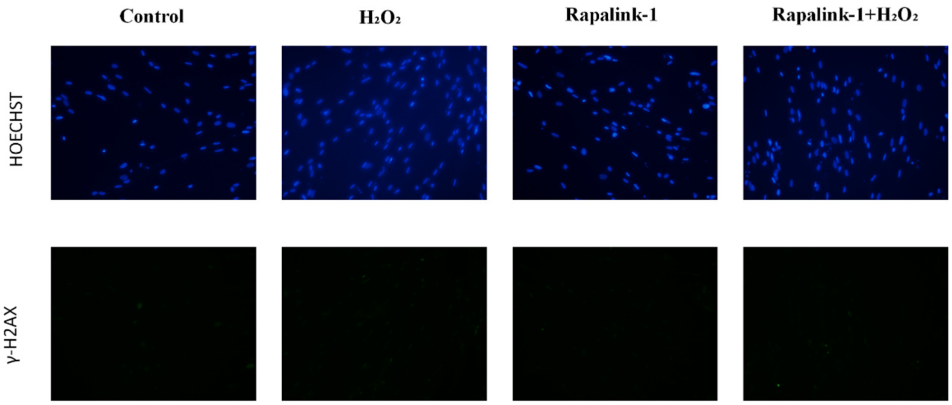

Figure S2 C 8-OHDG OF SMCS

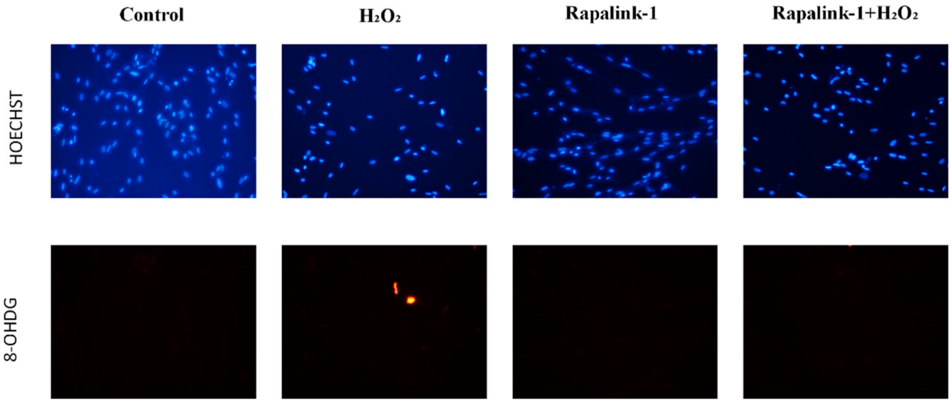

Representative immunofluorescence images corresponding to Figure 3A, C

Figure 3 β-Gal OF HUVECS

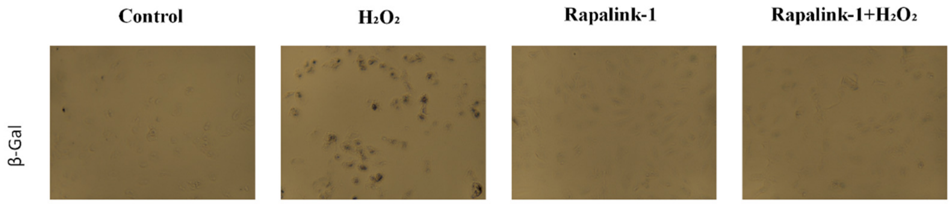

Figure 3 Lamin B1 OF HUVECS

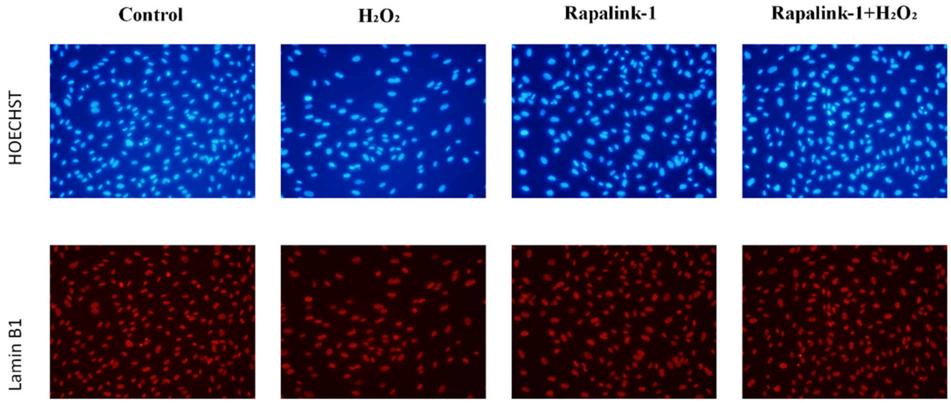

Representative immunofluorescence images corresponding to Supplementary Figure S3A

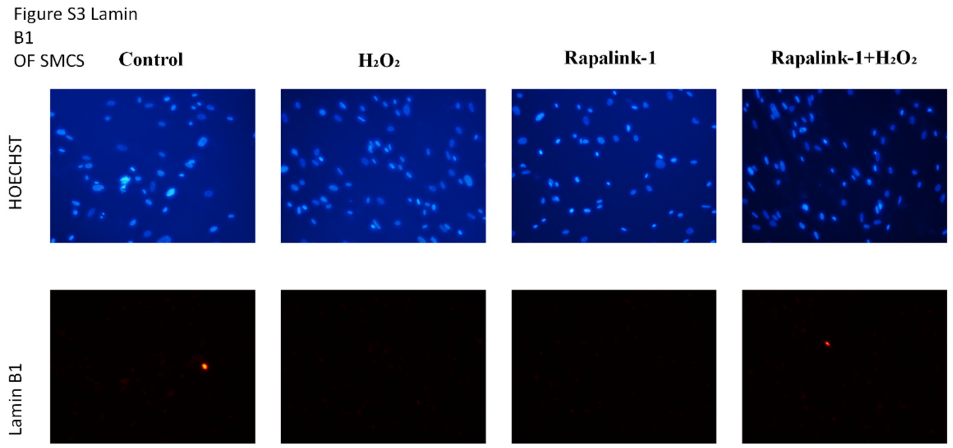

Uncropped WB corresponding to Figure 3E, Supplementary Figure S3C

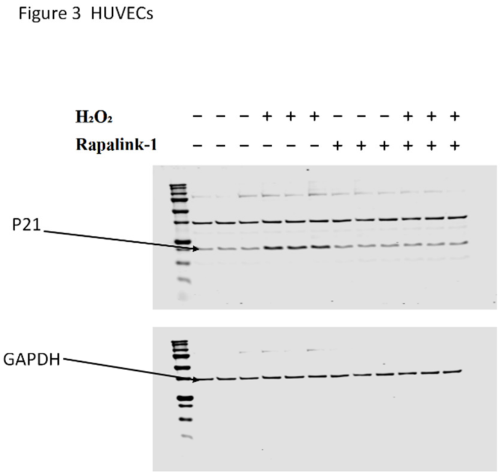

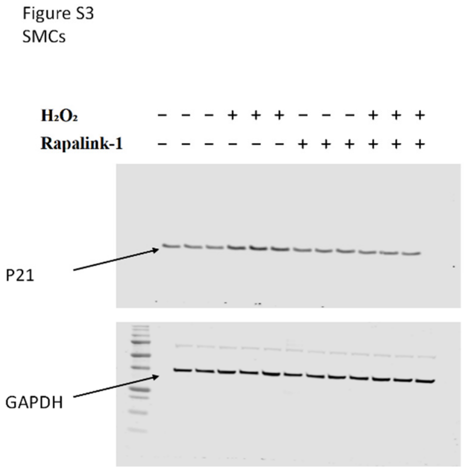

Uncropped WB corresponding to Figure 4E, Supplementary Figure S4E

Figure 4 WB OF HUVECs

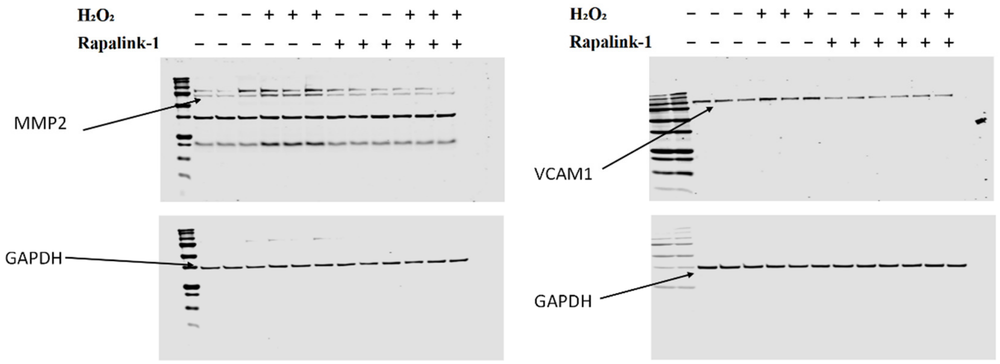

Figure S4 WB OF SMCs

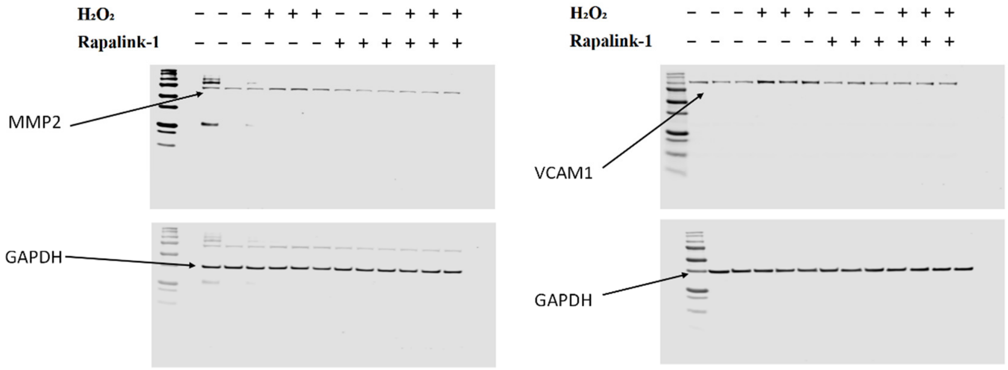

Uncropped WB corresponding to Figure 5A, Supplementary Figure S5A

Figure 5
WB OF
HUVECS

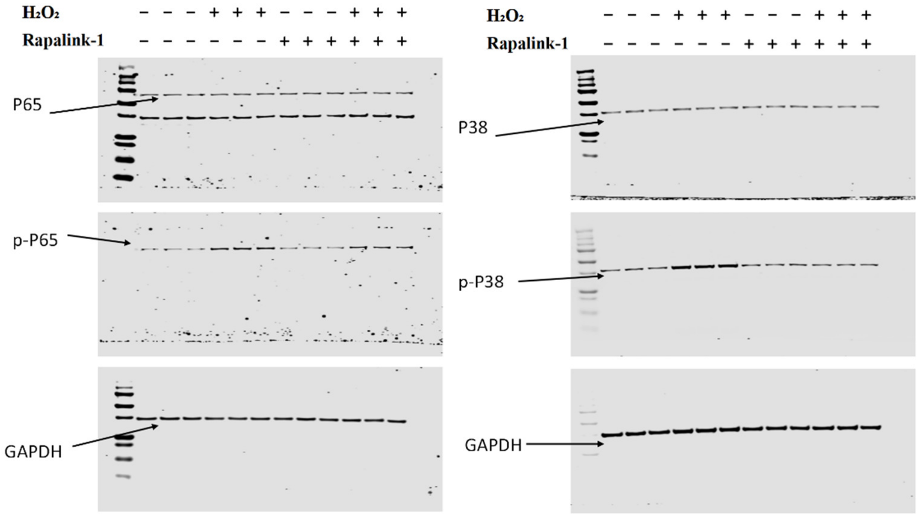

Figure 5
WB OF
HUVECS

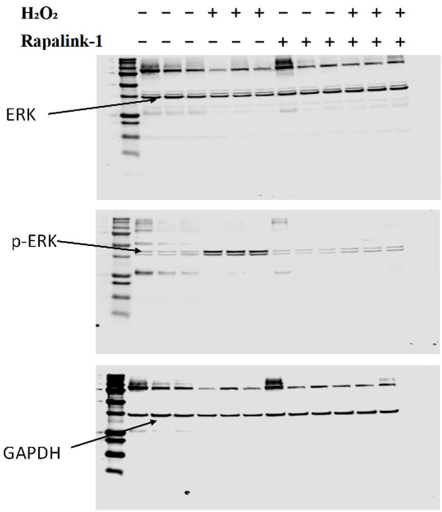

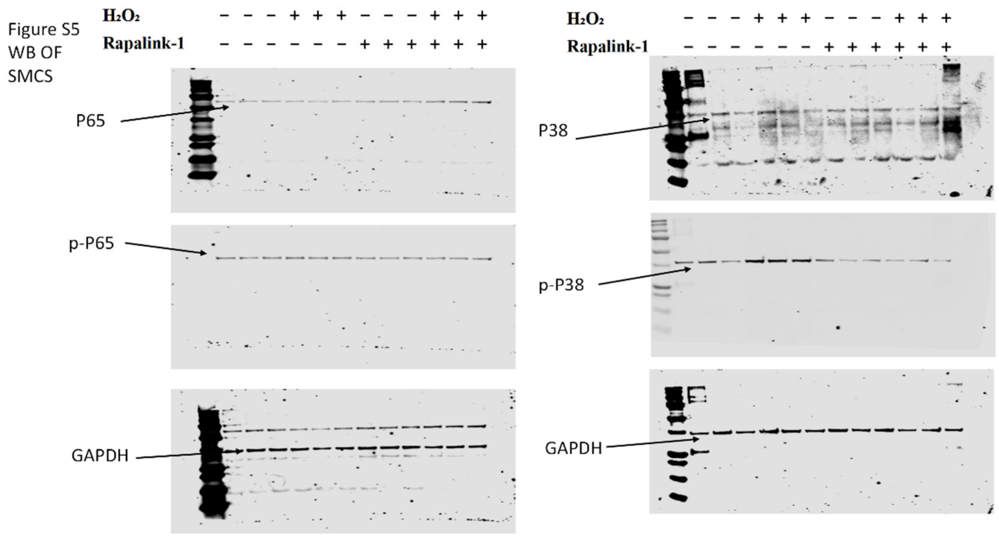

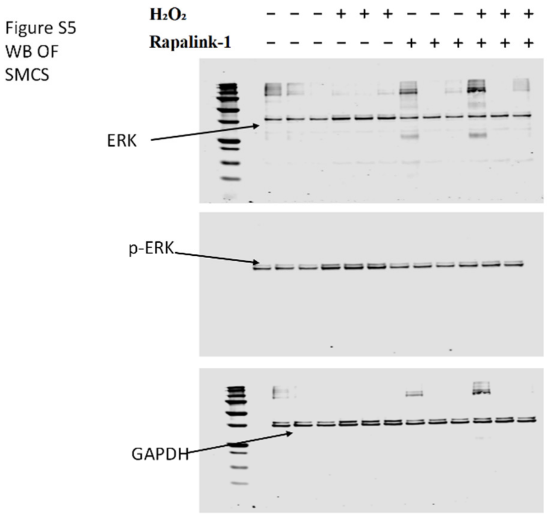

Uncropped WB corresponding to Figure 6A, Supplementary Figure S6A

Figure 6
WB OF
HUVECS

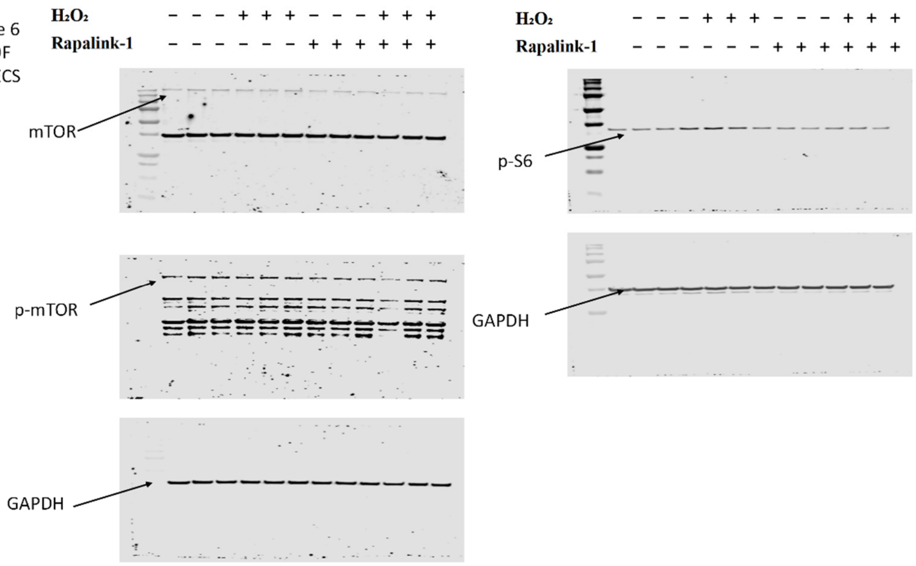

Figure 6
WB OF
HUVECS

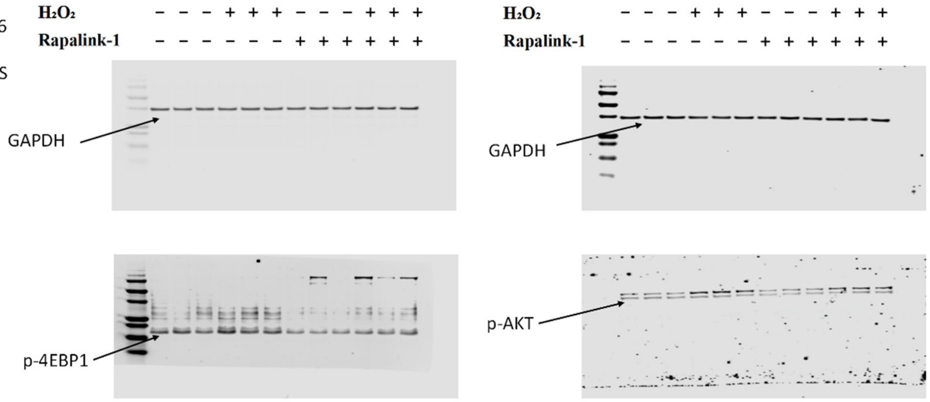

Figure S6
WB OF
SMCS

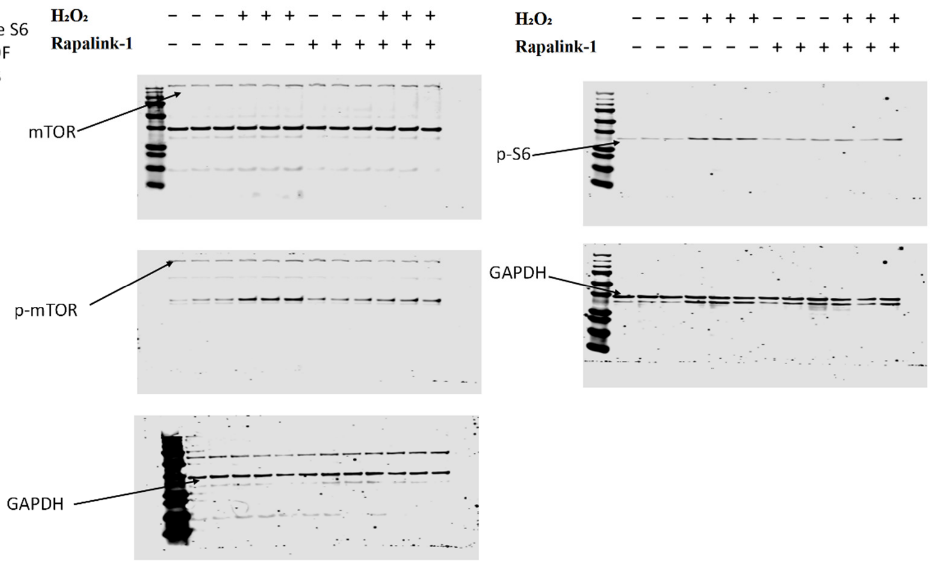

Figure S6
WB OF
SMCs

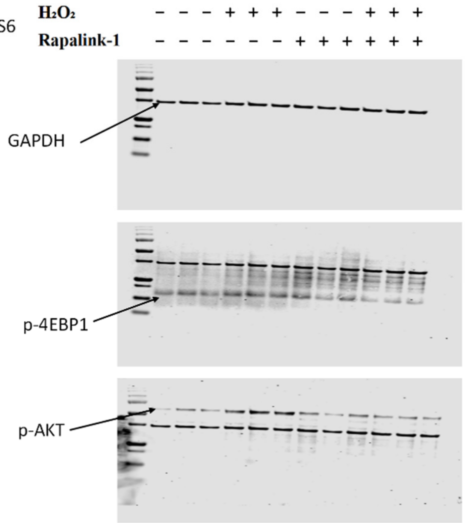

Supplement: Supplementary file 1 [file biology-15-00732-s001.zip › Supplementary Raw Data.pdf]
