# Supplementary material for: Rapalink-1 Attenuates Oxidative-Stress-Induced Senescence in Vascular Cells in Association with Reduced NF-κB and MAPK Signaling
Source: Biology (Basel). 2026 May 6;15(9):732. doi: 10.3390/biology15090732 (PMC13162792; doi:10.3390/biology15090732)
Supplement: Supplementary file 1 [file biology-15-00732-s001.zip › Supplementary Tables.pdf]

Supplementary Table S1 (Primary and Secondary antibodies)

Antibody	MW(kDa)	Brand	Catalog Number	Concentration
**P21**	**21**	**Cell Signaling**	**# 2947S**	**1:1,000**
**MMP2**	**70**	**Invitrogen**	**#436000**	**2 μg/mL**
**VCAM1**	**95–110**	**Abcam**	**ab174279**	**1:1,000**
**Lamin B1**	**66**	**Abcam**	**ab16048**	**0.1μg/mL**
**8-OHDG**		**BIOSS**	**BSS-BS-1278R**	**1:500**
**γ-H2AX**		**Cell Signaling**	**#80312S**	**1:400**
**P65**	**65**	**Cell Signaling**	**#6956S**	**1:1,000**
**p-P65**	**65**	**Cell Signaling**	**#3033S**	**1:1,000**
**p-P38**	**43**	**Cell Signaling**	**#4511S**	**1:1,000**
**p-ERK**	**42,44**	**Cell Signaling**	**#4370S**	**1:2,000**
**mTOR**	**289**	**Cell Signaling**	**#4517S**	**1:1,000**
**p-mTOR**	**289**	**Cell Signaling**	**#2971S**	**1:1,000**
**p-S6**	**32**	**Cell Signaling**	**#2215S**	**1:1,000**
**S6**	**32**	**Cell Signaling**	**#2217S**	**1:1,000**
**p-AKT**	**60**	**Cell Signaling**	**#9271S**	**1:1,000**
**p-4EBP1**	**15-20**	**Cell Signaling**	**#2855S**	**1:1,000**
**GAPDH (Mouse)**	**45**	**Invitrogen**	**#MA5-15739**	**1:1,000**
**Alexa Fluor 594**		**Cell Signaling**	**#8889S**	**1:10,000**
**Alexa Fluor 488**		**Cell Signaling**	**#4408S**	**1:10,000**

**IRDye 800CW Goat-anti-Rabbit Antibody**	**LI-COR**	**#926-32211**	**1:10,000**
**IRDye 680RD Donkey anti-Mouse IgG Secondary Antibody**	**LI-COR**	**#926-68072**	**1:10,000**

Supplementary Table S2 (qPCR primers)

**Target gene**	**Gene Accession Number**	**Sense 5′ -3′**	**Antisense 5′ -3′**
**NRF2**	**NM_006164**	**CAGCGACGGAAAGAGTATGA**	**TGGGCAACCTGGGAGTAG**
**NOX4**	**NM_016931**	**GCTGGATGGAGTGTGGAGAC**	**CAGGGTGGGTGAGGAAGAGT**
**MnSOD**	**NM_000636**	**AAGGGAGATGTTACAGCCCAGATA**	**TCCAGAAAATGCTATGATTGAATGAC**
**P16**	**NM_000077**	**CAACGCACCGAATAGTTACG**	**AGCACCACCAGCGTGTC**
**COX2**	**NM_000963**	**TGAGTACCGCAAACGCTTCTC**	**TGGACGAGGTTTTTCCACCAG**

**IL-6**	**NM_001371096**	**CTGCAGGACATGACAACTCATC**	**ATCTGAGGTGCCCATGCTAC**
**IL-8**	**NM_001354840**	**TGCCAAGGAGTGCTAAAG**	**CTCCACAACCCTCTGCAC**
**ICAM1**	**NM_000201**	**CACAGTCACCTATGGCAACGA**	**TGGCTTCGTCAGAATCACGTT**
**MMP1**	**NM_002421**	**CAGAGATGAAGTCCGGTTTTTC**	**GGGGTATCCGTGTAGCACAT**
**TIMP1**	**NM_003254**	**TGGCTTCTGGCATCCTGTTGTTG**	**CGCTGGTATAAGGTGGTCTGGTTG**
